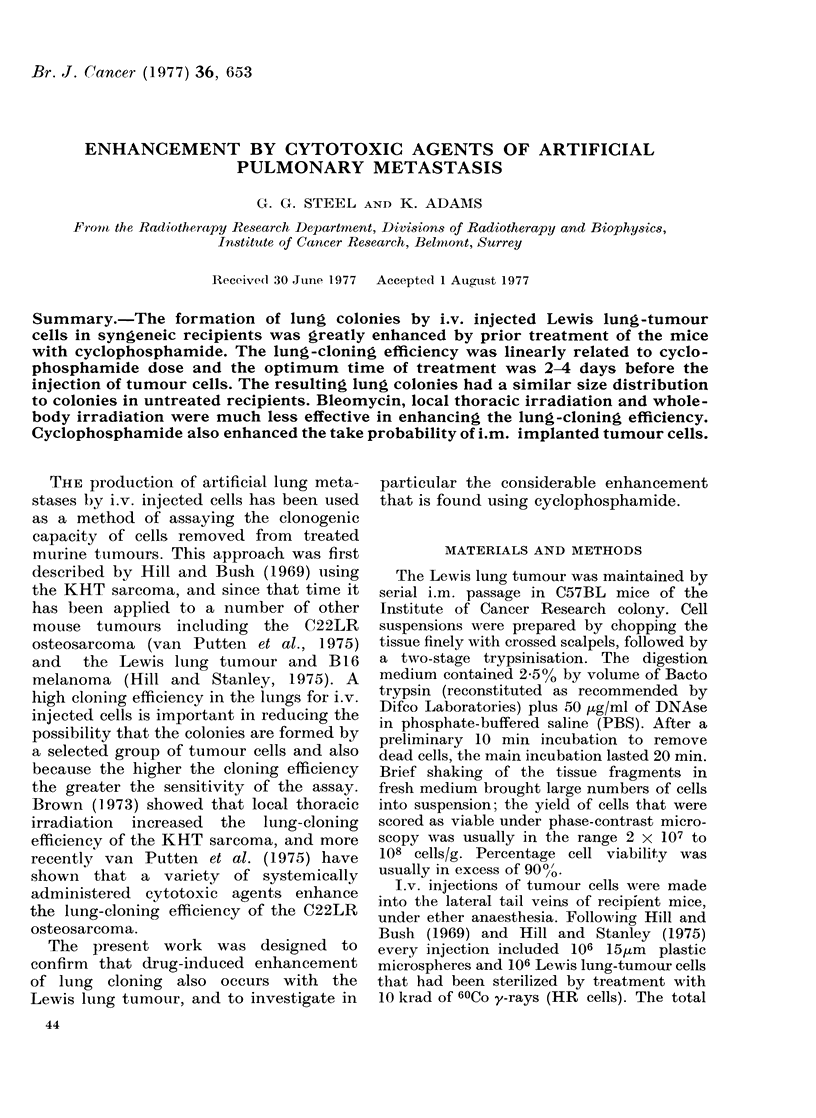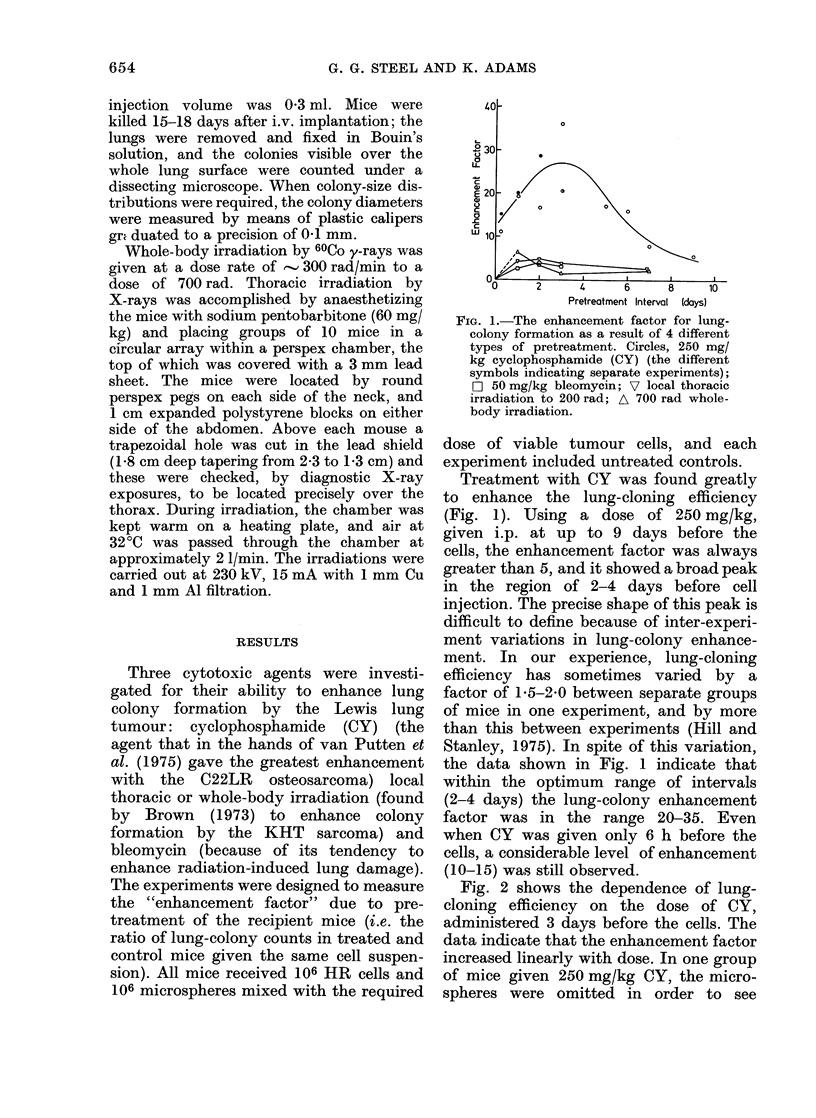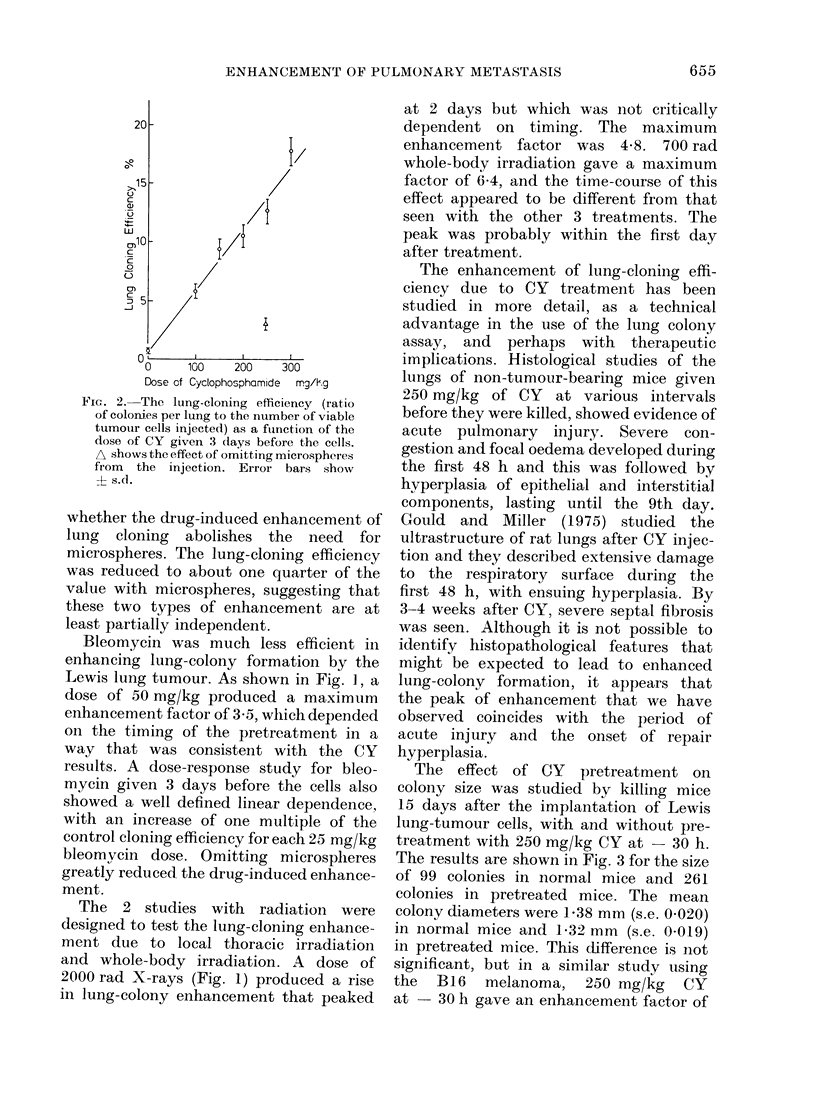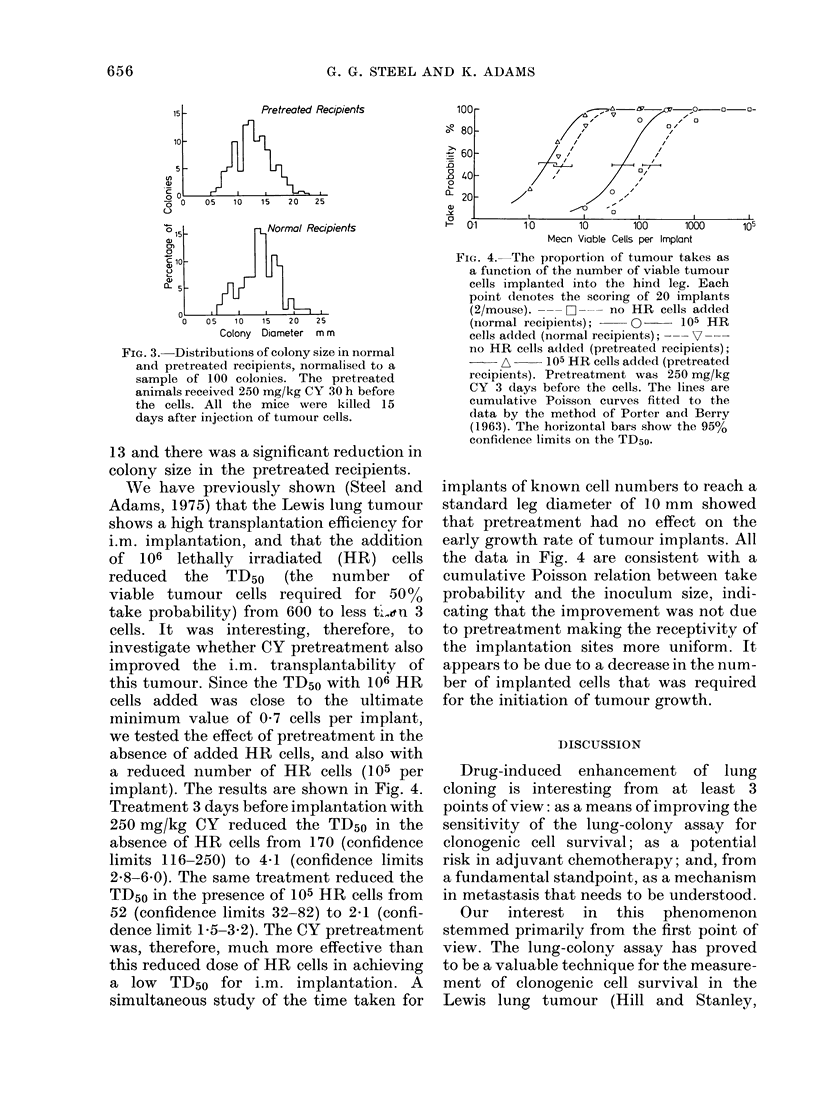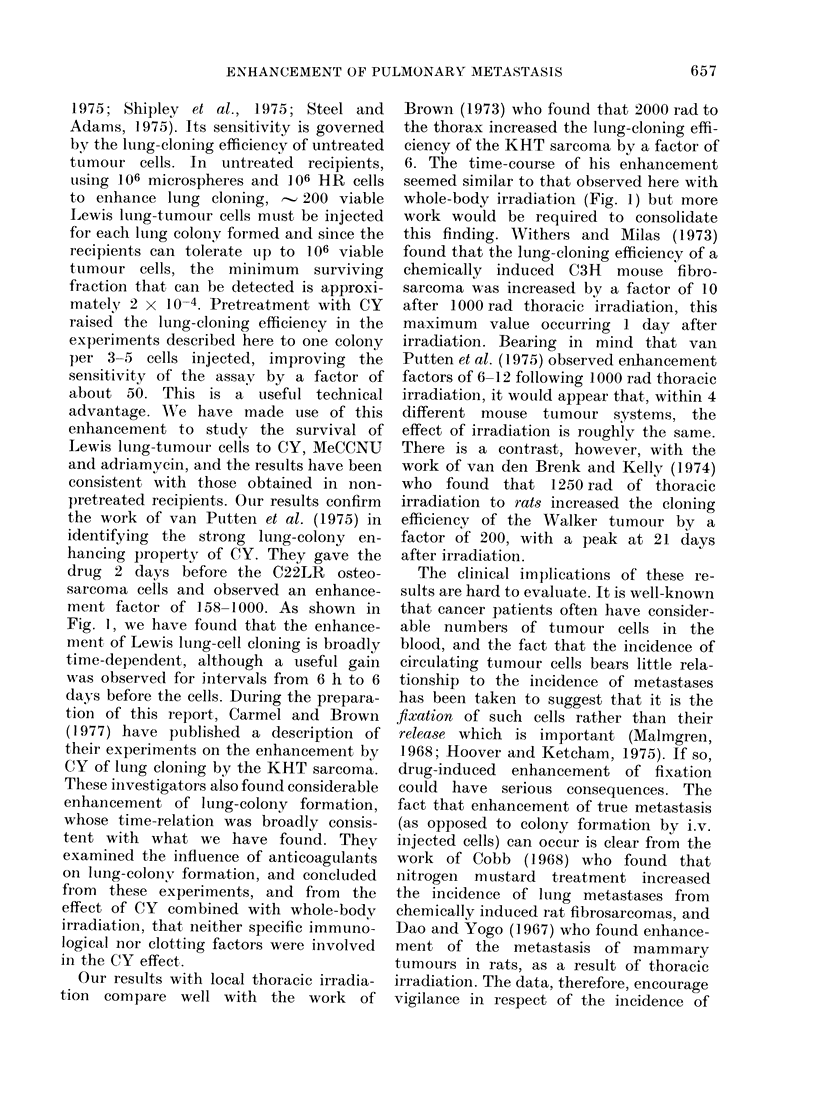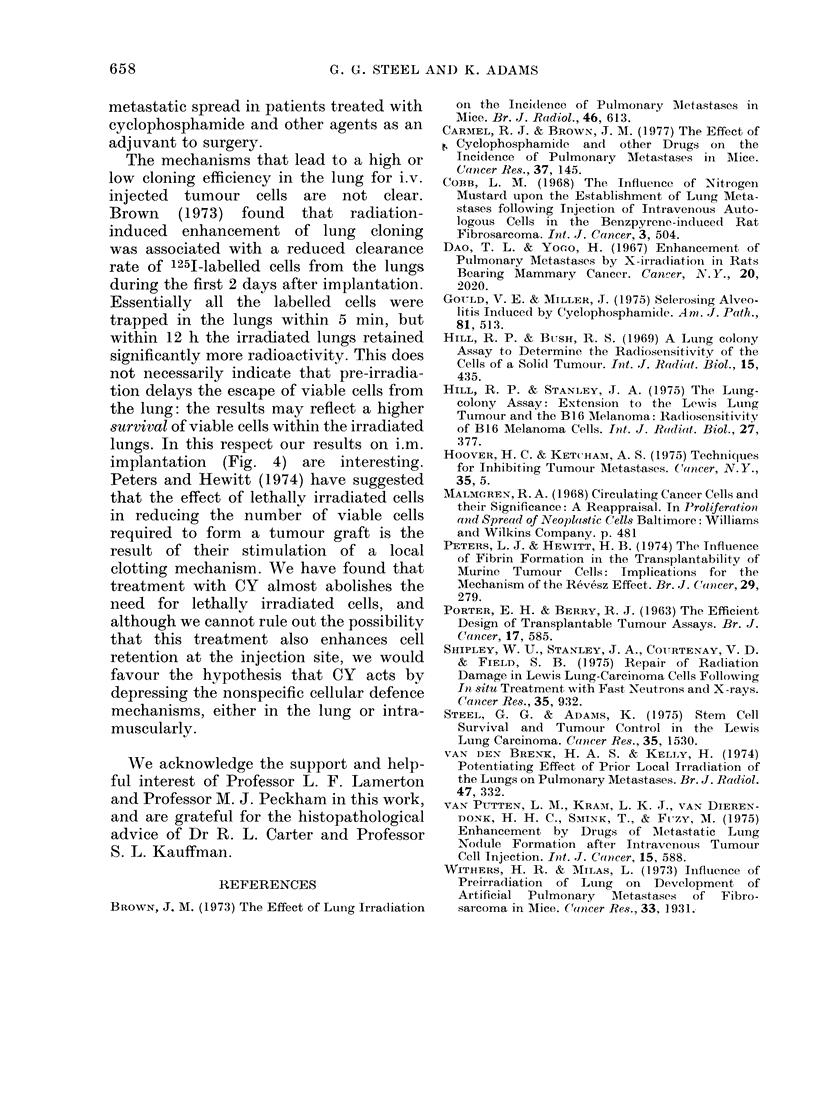# Enhancement by cytotoxic agents of artificial pulmonary metastasis.

**DOI:** 10.1038/bjc.1977.247

**Published:** 1977-12

**Authors:** G. G. Steel, K. Adams

## Abstract

The formation of lung colonies by i.v. injected Lewis lung-tumour cells in syngeneic recipients was greatly enhanced by prior treatment of the mice with cyclophosphamide. The lung-cloning efficiency was linearly related to cyclophosphamide dose and the optimum time of treatment was 2-4 days before the injection of tumour cells. The resulting lung colonies had a similar size distribution to colonies in untreated recipients. Bleomycin, local thoraric irradiation and whole-body irradiation were much less effective in enhancing the lung-cloning efficiency. Cyclophosphamide also enhanced the take probability of i.m. implanted tumour cells.


*Br. J. Cancer* (1977) **36**, 653

### ENHANCEMENT BY CYTOTOXIC AGENTS OF ARTIFICIAL PULMONARY METASTASIS

G. G. STEEL AND K. ADAMS

*From the Radiotherapy Research Department, Divisions of Radiotherapy and Biophysics, Institute of Cancer Research, Belmont, Surrey*

Received 30 June 1977 Accepted 1 August 1977

**Summary.—The formation of lung colonies by i.v. injected Lewis lung-tumour cells in syngeneic recipients was greatly enhanced by prior treatment of the mice with cyclophosphamide. The lung-cloning efficiency was linearly related to cyclophosphamide dose and the optimum time of treatment was 2–4 days before the injection of tumour cells. The resulting lung colonies had a similar size distribution to colonies in untreated recipients. Bleomycin, local thoracic irradiation and whole-body irradiation were much less effective in enhancing the lung-cloning efficiency. Cyclophosphamide also enhanced the take probability of i.m. implanted tumour cells.**

THE production of artificial lung metastases by i.v. injected cells has been used as a method of assaying the clonogenic capacity of cells removed from treated murine tumours. This approach was first described by Hill and Bush (1969) using the KHT sarcoma, and since that time it has been applied to a number of other mouse tumours including the C22LR osteosarcoma (van Putten *et al.*, 1975) and the Lewis lung tumour and B16 melanoma (Hill and Stanley, 1975). A high cloning efficiency in the lungs for i.v. injected cells is important in reducing the possibility that the colonies are formed by a selected group of tumour cells and also because the higher the cloning efficiency the greater the sensitivity of the assay. Brown (1973) showed that local thoracic irradiation increased the lung-cloning efficiency of the KHT sarcoma, and more recently van Putten *et al.* (1975) have shown that a variety of systemically administered cytotoxic agents enhance the lung-cloning efficiency of the C22LR osteosarcoma.

The present work was designed to confirm that drug-induced enhancement of lung cloning also occurs with the Lewis lung tumour, and to investigate in particular the considerable enhancement that is found using cyclophosphamide.

### MATERIALS AND METHODS

The Lewis lung tumour was maintained by serial i.m. passage in C57BL mice of the Institute of Cancer Research colony. Cell suspensions were prepared by chopping the tissue finely with crossed scalpels, followed by a two-stage trypsinisation. The digestion medium contained 2·5% by volume of Bacto trypsin (reconstituted as recommended by Difco Laboratories) plus 50 $\mu$g/ml of DNAse in phosphate-buffered saline (PBS). After a preliminary 10 min incubation to remove dead cells, the main incubation lasted 20 min. Brief shaking of the tissue fragments in fresh medium brought large numbers of cells into suspension; the yield of cells that were scored as viable under phase-contrast microscopy was usually in the range $2 \times 10^7$ to $10^8$ cells/g. Percentage cell viability was usually in excess of 90%.

I.v. injections of tumour cells were made into the lateral tail veins of recipient mice, under ether anaesthesia. Following Hill and Bush (1969) and Hill and Stanley (1975) every injection included $10^6$ 15$\mu$m plastic microspheres and $10^6$ Lewis lung-tumour cells that had been sterilized by treatment with 10 krad of $^{60}$Co $\gamma$-rays (HR cells). The total injection volume was 0·3 ml. Mice were killed 15–18 days after i.v. implantation; the lungs were removed and fixed in Bouin's solution, and the colonies visible over the whole lung surface were counted under a dissecting microscope. When colony-size distributions were required, the colony diameters were measured by means of plastic calipers graduated to a precision of 0·1 mm.

Whole-body irradiation by $^{60}$Co $\gamma$-rays was given at a dose rate of $\sim$300 rad/min to a dose of 700 rad. Thoracic irradiation by X-rays was accomplished by anaesthetizing the mice with sodium pentobarbitone (60 mg/kg) and placing groups of 10 mice in a circular array within a perspex chamber, the top of which was covered with a 3 mm lead sheet. The mice were located by round perspex pegs on each side of the neck, and 1 cm expanded polystyrene blocks on either side of the abdomen. Above each mouse a trapezoidal hole was cut in the lead shield (1·8 cm deep tapering from 2·3 to 1·3 cm) and these were checked, by diagnostic X-ray exposures, to be located precisely over the thorax. During irradiation, the chamber was kept warm on a heating plate, and air at 32°C was passed through the chamber at approximately 2 l/min. The irradiations were carried out at 230 kV, 15 mA with 1 mm Cu and 1 mm Al filtration.

### RESULTS

Three cytotoxic agents were investigated for their ability to enhance lung colony formation by the Lewis lung tumour: cyclophosphamide (CY) (the agent that in the hands of van Putten *et al.* (1975) gave the greatest enhancement with the C22LR osteosarcoma) local thoracic or whole-body irradiation (found by Brown (1973) to enhance colony formation by the KHT sarcoma) and bleomycin (because of its tendency to enhance radiation-induced lung damage). The experiments were designed to measure the "enhancement factor" due to pretreatment of the recipient mice (*i.e.* the ratio of lung-colony counts in treated and control mice given the same cell suspension). All mice received $10^6$ HR cells and $10^6$ microspheres mixed with the required dose of viable tumour cells, and each experiment included untreated controls.

FIG. 1.—The enhancement factor for lung-colony formation as a result of 4 different types of pretreatment. Circles, 250 mg/kg cyclophosphamide (CY) (the different symbols indicating separate experiments); □ 50 mg/kg bleomycin; ▽ local thoracic irradiation to 200 rad; △ 700 rad whole-body irradiation.

Treatment with CY was found greatly to enhance the lung-cloning efficiency (Fig. 1). Using a dose of 250 mg/kg, given i.p. at up to 9 days before the cells, the enhancement factor was always greater than 5, and it showed a broad peak in the region of 2–4 days before cell injection. The precise shape of this peak is difficult to define because of inter-experiment variations in lung-colony enhancement. In our experience, lung-cloning efficiency has sometimes varied by a factor of 1·5–2·0 between separate groups of mice in one experiment, and by more than this between experiments (Hill and Stanley, 1975). In spite of this variation, the data shown in Fig. 1 indicate that within the optimum range of intervals (2–4 days) the lung-colony enhancement factor was in the range 20–35. Even when CY was given only 6 h before the cells, a considerable level of enhancement (10–15) was still observed.

Fig. 2 shows the dependence of lung-cloning efficiency on the dose of CY, administered 3 days before the cells. The data indicate that the enhancement factor increased linearly with dose. In one group of mice given 250 mg/kg CY, the microspheres were omitted in order to see

FIG. 2.—The lung-cloning efficiency (ratio of colonies per lung to the number of viable tumour cells injected) as a function of the dose of CY given 3 days before the cells. △ shows the effect of omitting microspheres from the injection. Error bars show ± s.d.

whether the drug-induced enhancement of lung cloning abolishes the need for microspheres. The lung-cloning efficiency was reduced to about one quarter of the value with microspheres, suggesting that these two types of enhancement are at least partially independent.

Bleomycin was much less efficient in enhancing lung-colony formation by the Lewis lung tumour. As shown in Fig. 1, a dose of 50 mg/kg produced a maximum enhancement factor of 3·5, which depended on the timing of the pretreatment in a way that was consistent with the CY results. A dose-response study for bleomycin given 3 days before the cells also showed a well defined linear dependence, with an increase of one multiple of the control cloning efficiency for each 25 mg/kg bleomycin dose. Omitting microspheres greatly reduced the drug-induced enhancement.

The 2 studies with radiation were designed to test the lung-cloning enhancement due to local thoracic irradiation and whole-body irradiation. A dose of 2000 rad X-rays (Fig. 1) produced a rise in lung-colony enhancement that peaked at 2 days but which was not critically dependent on timing. The maximum enhancement factor was 4·8. 700 rad whole-body irradiation gave a maximum factor of 6·4, and the time-course of this effect appeared to be different from that seen with the other 3 treatments. The peak was probably within the first day after treatment.

The enhancement of lung-cloning efficiency due to CY treatment has been studied in more detail, as a technical advantage in the use of the lung colony assay, and perhaps with therapeutic implications. Histological studies of the lungs of non-tumour-bearing mice given 250 mg/kg of CY at various intervals before they were killed, showed evidence of acute pulmonary injury. Severe congestion and focal oedema developed during the first 48 h and this was followed by hyperplasia of epithelial and interstitial components, lasting until the 9th day. Gould and Miller (1975) studied the ultrastructure of rat lungs after CY injection and they described extensive damage to the respiratory surface during the first 48 h, with ensuing hyperplasia. By 3–4 weeks after CY, severe septal fibrosis was seen. Although it is not possible to identify histopathological features that might be expected to lead to enhanced lung-colony formation, it appears that the peak of enhancement that we have observed coincides with the period of acute injury and the onset of repair hyperplasia.

The effect of CY pretreatment on colony size was studied by killing mice 15 days after the implantation of Lewis lung-tumour cells, with and without pretreatment with 250 mg/kg CY at − 30 h. The results are shown in Fig. 3 for the size of 99 colonies in normal mice and 261 colonies in pretreated mice. The mean colony diameters were 1·38 mm (s.e. 0·020) in normal mice and 1·32 mm (s.e. 0·019) in pretreated mice. This difference is not significant, but in a similar study using the B16 melanoma, 250 mg/kg CY at − 30 h gave an enhancement factor of

FIG. 3.—Distributions of colony size in normal and pretreated recipients, normalised to a sample of 100 colonies. The pretreated animals received 250 mg/kg CY 30 h before the cells. All the mice were killed 15 days after injection of tumour cells.

FIG. 4.—The proportion of tumour takes as a function of the number of viable tumour cells implanted into the hind leg. Each point denotes the scoring of 20 implants (2/mouse). ---□--- no HR cells added (normal recipients); ——○—— $10^5$ HR cells added (normal recipients); ---▽--- no HR cells added (pretreated recipients); ——△—— $10^5$ HR cells added (pretreated recipients). Pretreatment was 250 mg/kg CY 3 days before the cells. The lines are cumulative Poisson curves fitted to the data by the method of Porter and Berry (1963). The horizontal bars show the 95% confidence limits on the $TD_{50}$.

13 and there was a significant reduction in colony size in the pretreated recipients.

We have previously shown (Steel and Adams, 1975) that the Lewis lung tumour shows a high transplantation efficiency for i.m. implantation, and that the addition of $10^6$ lethally irradiated (HR) cells reduced the $TD_{50}$ (the number of viable tumour cells required for 50% take probability) from 600 to less than 3 cells. It was interesting, therefore, to investigate whether CY pretreatment also improved the i.m. transplantability of this tumour. Since the $TD_{50}$ with $10^6$ HR cells added was close to the ultimate minimum value of 0·7 cells per implant, we tested the effect of pretreatment in the absence of added HR cells, and also with a reduced number of HR cells ($10^5$ per implant). The results are shown in Fig. 4. Treatment 3 days before implantation with 250 mg/kg CY reduced the $TD_{50}$ in the absence of HR cells from 170 (confidence limits 116–250) to 4·1 (confidence limits 2·8–6·0). The same treatment reduced the $TD_{50}$ in the presence of $10^5$ HR cells from 52 (confidence limits 32–82) to 2·1 (confidence limit 1·5–3·2). The CY pretreatment was, therefore, much more effective than this reduced dose of HR cells in achieving a low $TD_{50}$ for i.m. implantation. A simultaneous study of the time taken for implants of known cell numbers to reach a standard leg diameter of 10 mm showed that pretreatment had no effect on the early growth rate of tumour implants. All the data in Fig. 4 are consistent with a cumulative Poisson relation between take probability and the inoculum size, indicating that the improvement was not due to pretreatment making the receptivity of the implantation sites more uniform. It appears to be due to a decrease in the number of implanted cells that was required for the initiation of tumour growth.

### DISCUSSION

Drug-induced enhancement of lung cloning is interesting from at least 3 points of view: as a means of improving the sensitivity of the lung-colony assay for clonogenic cell survival; as a potential risk in adjuvant chemotherapy; and, from a fundamental standpoint, as a mechanism in metastasis that needs to be understood.

Our interest in this phenomenon stemmed primarily from the first point of view. The lung-colony assay has proved to be a valuable technique for the measurement of clonogenic cell survival in the Lewis lung tumour (Hill and Stanley,

1975; Shipley *et al.*, 1975; Steel and Adams, 1975). Its sensitivity is governed by the lung-cloning efficiency of untreated tumour cells. In untreated recipients, using $10^6$ microspheres and $10^6$ HR cells to enhance lung cloning, $\sim$200 viable Lewis lung-tumour cells must be injected for each lung colony formed and since the recipients can tolerate up to $10^6$ viable tumour cells, the minimum surviving fraction that can be detected is approximately $2 \times 10^{-4}$. Pretreatment with CY raised the lung-cloning efficiency in the experiments described here to one colony per 3–5 cells injected, improving the sensitivity of the assay by a factor of about 50. This is a useful technical advantage. We have made use of this enhancement to study the survival of Lewis lung-tumour cells to CY, MeCCNU and adriamycin, and the results have been consistent with those obtained in non-pretreated recipients. Our results confirm the work of van Putten *et al.* (1975) in identifying the strong lung-colony enhancing property of CY. They gave the drug 2 days before the C22LR osteosarcoma cells and observed an enhancement factor of 158–1000. As shown in Fig. 1, we have found that the enhancement of Lewis lung-cell cloning is broadly time-dependent, although a useful gain was observed for intervals from 6 h to 6 days before the cells. During the preparation of this report, Carmel and Brown (1977) have published a description of their experiments on the enhancement by CY of lung cloning by the KHT sarcoma. These investigators also found considerable enhancement of lung-colony formation, whose time-relation was broadly consistent with what we have found. They examined the influence of anticoagulants on lung-colony formation, and concluded from these experiments, and from the effect of CY combined with whole-body irradiation, that neither specific immunological nor clotting factors were involved in the CY effect.

Our results with local thoracic irradiation compare well with the work of Brown (1973) who found that 2000 rad to the thorax increased the lung-cloning efficiency of the KHT sarcoma by a factor of 6. The time-course of his enhancement seemed similar to that observed here with whole-body irradiation (Fig. 1) but more work would be required to consolidate this finding. Withers and Milas (1973) found that the lung-cloning efficiency of a chemically induced C3H mouse fibrosarcoma was increased by a factor of 10 after 1000 rad thoracic irradiation, this maximum value occurring 1 day after irradiation. Bearing in mind that van Putten *et al.* (1975) observed enhancement factors of 6–12 following 1000 rad thoracic irradiation, it would appear that, within 4 different mouse tumour systems, the effect of irradiation is roughly the same. There is a contrast, however, with the work of van den Brenk and Kelly (1974) who found that 1250 rad of thoracic irradiation to *rats* increased the cloning efficiency of the Walker tumour by a factor of 200, with a peak at 21 days after irradiation.

The clinical implications of these results are hard to evaluate. It is well-known that cancer patients often have considerable numbers of tumour cells in the blood, and the fact that the incidence of circulating tumour cells bears little relationship to the incidence of metastases has been taken to suggest that it is the *fixation* of such cells rather than their *release* which is important (Malmgren, 1968; Hoover and Ketcham, 1975). If so, drug-induced enhancement of fixation could have serious consequences. The fact that enhancement of true metastasis (as opposed to colony formation by i.v. injected cells) can occur is clear from the work of Cobb (1968) who found that nitrogen mustard treatment increased the incidence of lung metastases from chemically induced rat fibrosarcomas, and Dao and Yogo (1967) who found enhancement of the metastasis of mammary tumours in rats, as a result of thoracic irradiation. The data, therefore, encourage vigilance in respect of the incidence of metastatic spread in patients treated with cyclophosphamide and other agents as an adjuvant to surgery.

The mechanisms that lead to a high or low cloning efficiency in the lung for i.v. injected tumour cells are not clear. Brown (1973) found that radiation-induced enhancement of lung cloning was associated with a reduced clearance rate of $^{125}$I-labelled cells from the lungs during the first 2 days after implantation. Essentially all the labelled cells were trapped in the lungs within 5 min, but within 12 h the irradiated lungs retained significantly more radioactivity. This does not necessarily indicate that pre-irradiation delays the escape of viable cells from the lung: the results may reflect a higher *survival* of viable cells within the irradiated lungs. In this respect our results on i.m. implantation (Fig. 4) are interesting. Peters and Hewitt (1974) have suggested that the effect of lethally irradiated cells in reducing the number of viable cells required to form a tumour graft is the result of their stimulation of a local clotting mechanism. We have found that treatment with CY almost abolishes the need for lethally irradiated cells, and although we cannot rule out the possibility that this treatment also enhances cell retention at the injection site, we would favour the hypothesis that CY acts by depressing the nonspecific cellular defence mechanisms, either in the lung or intramuscularly.

We acknowledge the support and helpful interest of Professor L. F. Lamerton and Professor M. J. Peckham in this work, and are grateful for the histopathological advice of Dr R. L. Carter and Professor S. L. Kauffman.

### REFERENCES

Brown, J. M. (1973) The Effect of Lung Irradiation on the Incidence of Pulmonary Metastases in Mice. *Br. J. Radiol.*, **46**, 613.

Carmel, R. J. & Brown, J. M. (1977) The Effect of Cyclophosphamide and other Drugs on the Incidence of Pulmonary Metastases in Mice. *Cancer Res.*, **37**, 145.

Cobb, L. M. (1968) The Influence of Nitrogen Mustard upon the Establishment of Lung Metastases following Injection of Intravenous Autologous Cells in the Benzpyrene-induced Rat Fibrosarcoma. *Int. J. Cancer*, **3**, 504.

Dao, T. L. & Yogo, H. (1967) Enhancement of Pulmonary Metastases by X-irradiation in Rats Bearing Mammary Cancer. *Cancer, N.Y.*, **20**, 2020.

Gould, V. E. & Miller, J. (1975) Sclerosing Alveolitis Induced by Cyclophosphamide. *Am. J. Path.*, **81**, 513.

Hill, R. P. & Bush, R. S. (1969) A Lung colony Assay to Determine the Radiosensitivity of the Cells of a Solid Tumour. *Int. J. Radiat. Biol.*, **15**, 435.

Hill, R. P. & Stanley, J. A. (1975) The Lung-colony Assay: Extension to the Lewis Lung Tumour and the B16 Melanoma: Radiosensitivity of B16 Melanoma Cells. *Int. J. Radiat. Biol.*, **27**, 377.

Hoover, H. C. & Ketcham, A. S. (1975) Techniques for Inhibiting Tumour Metastases. *Cancer, N.Y.*, **35**, 5.

Malmgren, R. A. (1968) Circulating Cancer Cells and their Significance: A Reappraisal. In *Proliferation and Spread of Neoplastic Cells* Baltimore: Williams and Wilkins Company. p. 481

Peters, L. J. & Hewitt, H. B. (1974) The Influence of Fibrin Formation in the Transplantability of Murine Tumour Cells: Implications for the Mechanism of the Révész Effect. *Br. J. Cancer*, **29**, 279.

Porter, E. H. & Berry, R. J. (1963) The Efficient Design of Transplantable Tumour Assays. *Br. J. Cancer*, **17**, 585.

Shipley, W. U., Stanley, J. A., Courtenay, V. D. & Field, S. B. (1975) Repair of Radiation Damage in Lewis Lung-Carcinoma Cells Following *In situ* Treatment with Fast Neutrons and X-rays. *Cancer Res.*, **35**, 932.

Steel, G. G. & Adams, K. (1975) Stem Cell Survival and Tumour Control in the Lewis Lung Carcinoma. *Cancer Res.*, **35**, 1530.

van den Brenk, H. A. S. & Kelly, H. (1974) Potentiating Effect of Prior Local Irradiation of the Lungs on Pulmonary Metastases. *Br. J. Radiol.* **47**, 332.

van Putten, L. M., Kram, L. K. J., van Dierendonk, H. H. C., Smink, T., & Fuzy, M. (1975) Enhancement by Drugs of Metastatic Lung Nodule Formation after Intravenous Tumour Cell Injection. *Int. J. Cancer*, **15**, 588.

Withers, H. R. & Milas, L. (1973) Influence of Preirradiation of Lung on Development of Artificial Pulmonary Metastases of Fibrosarcoma in Mice. *Cancer Res.*, **33**, 1931.